# Influence of Sonication on the Molecular Characteristics of Carbopol^®^ and Its Rheological Behavior in Microgels

**DOI:** 10.3390/gels10070420

**Published:** 2024-06-26

**Authors:** José Pérez-González, Yusef Muñoz-Castro, Francisco Rodríguez-González, Benjamín M. Marín-Santibáñez, Esteban F. Medina-Bañuelos

**Affiliations:** 1 Laboratorio de Reología y Física de la Materia Blanda, Escuela Superior de Física y Matemáticas, Instituto Politécnico Nacional, U. P. Adolfo López Mateos, Ciudad de México C.P. 07738, Mexico; yusefmunoz@gmail.com; 2 Departamento de Biotecnología, Centro de Desarrollo de Productos Bióticos, Instituto Politécnico Nacional, Carretera Yautepec-Jojutla Km. 6, Calle CEPROBI No. 8, Col. San Isidro, Yautepec, Morelos C.P. 62731, Mexico; frrodriguezg@ipn.mx; 3 Escuela Superior de Ingeniería Química e Industrias Extractivas, Instituto Politécnico Nacional, U. P. Adolfo López Mateos, Ciudad de México C.P. 07738, Mexico; bmarin@ipn.mx (B.M.M.-S.); efmedinab1400@alumno.ipn.mx (E.F.M.-B.)

**Keywords:** Carbopol^®^ Ultrez 10 microgels, rheology, yield-stress fluids, ultrasonication, Fourier transform infrared (FTIR) spectroscopy, confocal microscopy

## Abstract

In this work, the effect of sonication on the molecular characteristics of polyacrylic acid (Carbopol^®^ Ultrez 10), as well as on its rheological behavior in aqueous dispersions and microgels, was analyzed for the first time by rheometry, weight-average molecular weight (*M_w_*) measurements via static light scattering (SLS), Fourier transform infrared (FTIR) spectroscopy and confocal microscopy. For this, the precursor dispersion and the microgels containing 0.25 wt.% of Ultrez 10 were sonicated in a commercial ultrasound bath at constant power and at different times. The main rheological properties of the microgel, namely, shear modulus, yield stress and viscosity, all decreased with increasing sonication time, while the microgel’s Herschel–Bulkley (H-B) behavior, without thixotropy, was preserved. Also, *M_w_* of Ultrez 10 decreased up to almost one-third (109,212 g/mol) of its original value (300,860 g/mol) after 180 min of sonication. These results evidence a softening of the gel microstructure, which results from the reduction in the *M_w_* of polyacrylic acid with sonication time. Separately, FTIR measurements show that sonication produces scission in the C-C links of the Carbopol^®^ backbone, which results in chains with the same chemistry but lower molecular weight. Finally, confocal microscopy observations revealed a diminution of the size of the microsponge domains and more free solvent with sonication time, which is reflected in a less compact and softer microstructure. The present results indicate that both the microstructure and the rheological behavior of Carbopol^®^ microgels, in particular, and complex fluids, in general, may be manipulated or tailored by systematic high-power ultrasonication.

## 1. Introduction

Sonication or ultrasonication is the application of ultrasound energy to a sample, which most often consists of a fluid with dispersed particles. There are two main ways for sonication, namely, by using an ultrasonic bath or a probe sonicator. In the first mode, the fluid in a vessel is set in a water-containing ultrasonic bath; in the second, the ultrasonic probe is immersed in the fluid of interest. Applications of ultrasound may be roughly divided into low-power (<1 W/cm^2^) and high-frequency (>100 kHz) regimes, as well as in high-power (>1 W/cm^2^) and low-frequency (20–100 kHz) regimes, being the first mainly used for non-destructive analysis or materials characterization, while the second is used in industrial processes as well as to produce chemical reactions and changes in the microstructure of materials [1,2].

According to the Royal Society of Chemistry [3], propagation of ultrasonic waves (typically >20 kHz) in a liquid medium results in agitation along with alternating high-pressure (compression) and low-pressure (rarefaction) cycles. During rarefaction, high-intensity sonic waves create small vacuum bubbles or voids in the liquid, which then collapse violently (cavitation) during compression, creating very high local temperatures and stresses. Thus, prolonged high-intensity sonication may produce chemical reactions in a sample. High-intensity sonication has been exploited in many applications, including cleaning, drilling, soldering, chemical processes, emulsification, deagglomeration, extraction, cell disruption [4], dispersion of nanoparticles in nanofluids [5] and others, as those found in food science and processing [6,7,8].

An application of sonication of particular interest to this work is the possible manipulation or tailoring of the microstructure of complex fluids, including gels. Gels appear in many everyday products, such as cosmetics, pharmaceuticals, detergents, coatings and foods, among many others. Therefore, tuning the flow or rheological properties of gels using ultrasound may be of practical relevance. Interestingly, scarce work has been conducted to understand the gel structural changes and its concomitant rheological behavior arising from sonication. In this regard, Seshadri et al. [9] studied the effect of high-intensity ultrasound (40 W) at various times on the rheological and optical properties of high-methoxyl pectin (HMP) dispersions. These authors found that ultrasonically pretreated pectin dispersions formed weaker gels with increasing sonication power and time and resulted in more transparent gels. The results were attributed to an overall reduction in the average molecular weight of pectin due to cavitational effects. Rajabali et al. [10] reported degradation of hydrogels made from acrylic acid and acrylamide; in this case, degradation was inferred from a decrease in the viscosity of the hydrogel with sonication time. Later, Prajapat and Gogate [11] reported depolymerization of a polyacrylic acid solution submitted to ultrasound irradiation. In this case, depolymerization was deduced from a reduction in the intrinsic viscosity of the solution with sonication time. Zheng et al. [12] also analyzed the effects of sonication at different powers (120, 240, 360 and 480 W) on the rheological properties of HMP dispersions and showed that their viscosity was reduced significantly with increasing sonication power and time; meanwhile, the overall pseudoplastic behavior of the gel was retained. Zheng et al. [12] suggested that the cavitation effect damaged the structure of HMP as ultrasonic power increased, leading to a significantly decreased strength of the gel.

Recently, Gibaud et al. [13] introduced what they called “rheoacoustic” gels; that is, colloidal gels sensitive to ultrasonic vibrations. These authors used a combination of rheological and structural characterization to evidence and quantify a strong softening, including decreased yield stress and accelerated shear-induced fluidization, in three different colloidal gels submitted to ultrasonic vibrations (with submicron amplitude and frequencies in the range between 20 and 500 kHz). The softening was attributed to micron-sized cracks within the gel network, which could or could not fully heal, depending on the acoustic intensity, once vibrations are turned off.

The purpose of this work is twofold; the first one is to understand the effects of high-power sonication on the molecular characteristics of Carbopol^®^ and its rheological behavior in microgels. The second is to explore the possibility of manipulation or tailoring the microstructure of Carbopol^®^ and its rheological behavior in dispersions and microgels in particular, as well as complex fluids, in general, by systematic high-power ultrasound irradiation. Carbopol^®^ polymers are used in a variety of applications encompassing the cosmetics, pharmaceutical, paint and food industries as a thickening, gelling, suspending, dispersing and stabilizing agent. Thus, the effects of sonication time at a fixed power on the molecular characteristics of Ultrez 10 and its rheological behavior in a 0.25 wt.% dispersion in bi-distilled water and the resulting microgel after neutralization were analyzed by rheometry, molecular weight measurements via static light scattering (SLS), Fourier transform infrared (FTIR) spectroscopy and confocal microscopy. The precursor dispersion and the microgel were sonicated in a commercial ultrasound bath at constant power at various times. We observed a softening of the microgel microstructure consisting of a systematic decrease in its shear modulus, yield stress and viscosity with increasing sonication time, while the Herschel–Bulkley behavior was maintained. SLS measurements evidenced a reduction in *M_w_* of polyacrylic acid with sonication time. Separately, FTIR measurements indicate that sonication produces scission in the C-C links of the Ultrez 10 backbone, which results in chains with the same chemistry but lower molecular weight. Finally, confocal microscopic measurements revealed a concomitant diminution of the size of the microsponge domains resulting from a reduction in the molecular weight of polyacrylic acid with sonication time. Overall, results in this work indicate that both the microstructure and rheological behavior of microgels, in particular, and complex fluids in general, may be manipulated or tailored by high-power ultrasonication. Special interest may be paid to naturally occurring or synthetic polymers whose high molecular weight results in high-viscosity solutions and gels that limit their applications.

## 2. Results and Discussion

### 2.1. Rheological Behavior of Non-Sonicated and Sonicated Microgels: Small Amplitude Oscillatory Shear Measurements

To assess the changes in rheological behavior of the Ultrez 10 microgels due to ultrasound treatment, we performed oscillatory shear and rotational steady shear measurements. We first discuss the viscoelastic response in small amplitude oscillatory shear (SAOS) measurements and, afterward, the steady shear behavior of the microgels.

**Figure 1:**
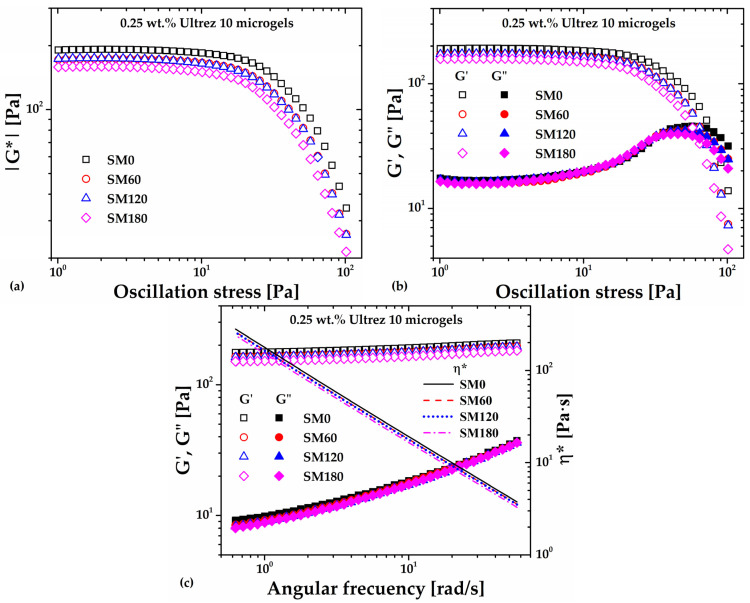
SAOS measurements of the Ultrez 10 microgel submitted to different sonication times: (**a**) |G*| as a function of the oscillating stress; (**b**) *G′* and *G″* as functions of the oscillating stress, at 6.28 rad/s; (**c**) *G′*, *G″* and η* as functions of the angular frequency at 3 Pa.

Figure 1a shows the absolute value of the complex shear modulus (|G*|) measured in stress amplitude sweeps at an angular frequency (*ω*) of 6.28 rad/s for the non-sonicated, SM0, and sonicated microgels samples, namely, SM60, SM120 and SM180 (the number in the labels represents the sonication time in minutes). Clearly, |G*| decreases with increasing the sonication time; its value in the linear viscoelastic region (LVR) decreases ~25%, from about 192 Pa for SM0 up to 159 Pa for SM180, evidencing a weakening or softening of the microgel microstructure due to the imposed ultrasound irradiation. Figure 1b indicates that the softening of the microgel microstructure can be attributed to a decrease in its elastic response or elastic modulus (*G′*) since the loss modulus (*G″*) barely changes in the LVR with increasing the sonication time. In addition, the limit of the LVR and the crossover point between *G′* and *G″* decrease with increasing sonication time, which indicates that ultrasound treatment promotes anticipated fluidization. Lastly, Figure 1c shows the frequency sweep for the microgel with different sonication times (the complex viscosity η* is included for completeness). Note that the value of the *G′*/*G″* ratios for different sonication times is of the order of ten or higher in the range of frequency analyzed, indicating that the 0.25 wt.% Ultrez 10 microgel remains a strong gel [14] for the periods of irradiation in this work. On its side, η*, which is a more representative parameter for fluid-like samples and indicates the overall resistance to shear deformation, decreases systematically with sonication time.

### 2.2. Rheological Behavior of Non-Sonicated and Sonicated Microgels: Steady Shear Measurements

**Figure 2:**
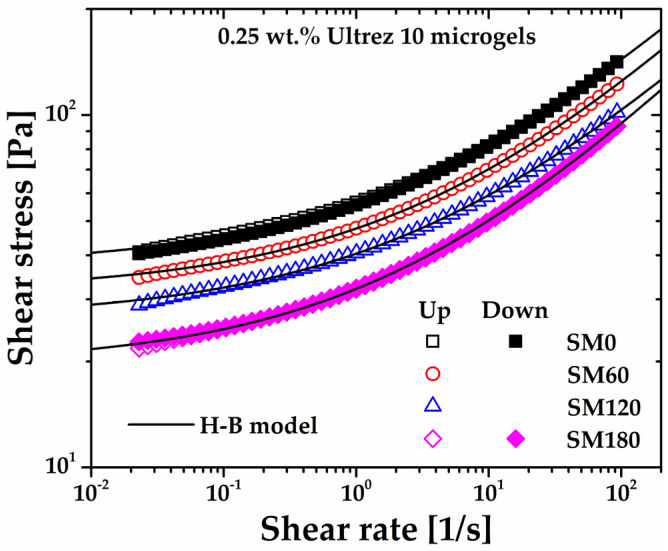
Steady shear flow curves of the Ultrez 10 microgel for different sonication times. The flow curves for SM0 and SM180 obtained in up and down shear stress cycles are included as proof of the absence of thixotropy.

The flow curves of the microgel for different sonication times obtained under steady shear measurements are shown in Figure 2, which includes the flow curves for SM0 and SM180 obtained in up and down shear stress cycles. First, it can be seen that SM0 and SM180 up and down flow curves superpose very well, respectively, indicating that the microgel is originally non-thixotropic [15,16] and that sonication does not induce thixotropy. The same behavior was observed for all up and down flow curves (these are not included in Figure 2 so as not to make the plot heavy). Also, as the share rate tends to zero, all flow curves extrapolate to a critical shear stress value, i.e., the yield stress of the microgels. Irrespective of the sonication time, they are all very well described by the H-B constitutive model, $\sigma =\sigma_{y}+m{\dot{\gamma }}^{n}$, where *σ* is the shear stress, *σ_y_* is the yield stress, *m* is the consistency index, $\dot{\gamma }$ is the shear rate, and *n* is the shear rate sensitivity index, in agreement with previous reports for similar non-sonicated microgels [15,16,17,18,19,20]. The H-B parameters for the microgels with different sonication times appear in Table 1.

**Figure 3:**
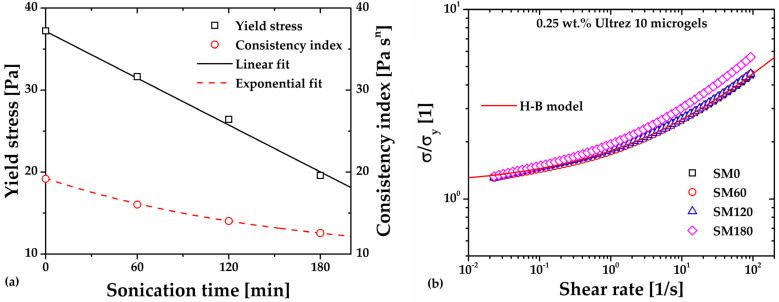
(**a**) Consistency index and yield stress of Ultrez 10 microgel as functions of sonication time. (**b**) *σ*/*σ_y_* as a function of the shear rate.

A significant decrease is observed, however, in the consistency index and yield stress of the microgel, that is, the first in an exponential way and the second linearly with increasing sonication time (Figure 3a). Regarding the power–law or shear-thinning index of the microgel, this remains almost constant, with a more significant variation for SM180. This anomalous behavior is evidenced in Figure 3b, where the different flow curves have been superposed by plotting *σ*/*σ_y_* as a function of the shear rate (see references [14,17,19]). Note that the SM180 flow curve departs from the superposed ones, indicating a dramatic change in the gel microstructure at long sonication times. Gutowski et al. [18] interpreted different scaling among low- and high-concentration Ultrez 10 microgels as a signal of a meaningful change in the mesostructure of the materials, which agrees with the result in this work (see discussion in Section 2.5 below).

The softening of the microgel microstructure and its nearly constant pseudoplastic behavior upon submission to sonication is consistent with previous reports for other gel systems [10,12,13]. From X-ray scattering measurements in their colloidal gels during sonication, Gibaud et al. [13] attributed this softening to micron-sized cracks within the gel network; these authors concluded that the gel network is fractured by ultrasonic vibrations and suggested that a more complete picture of the gel microstructure remained to be obtained. In Section 2.4 and Section 2.5, we provide such a picture based on measurements of changes in the molecular characteristics of Ultrez 10 after sonication and confocal microscopy.

### 2.3. Rheometry of the Sonicated Ultrez 10 Dispersion and Its Microgel

**Figure 4:**
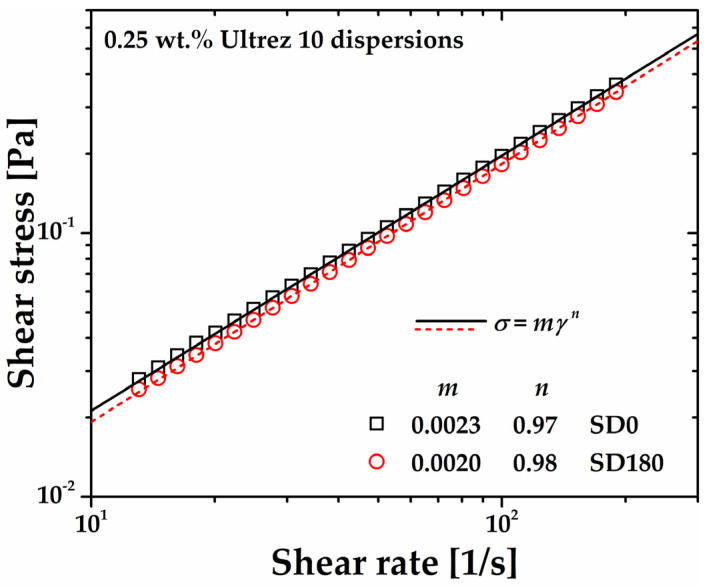
Steady-state flow curves of the non-sonicated (SD0) and sonicated (SD180) 0.25 wt.% Ultrez 10 dispersions.

In a different experiment to assess ultrasound irradiation-induced damage of the molecular structure of polyacrylic acid, we sonicated a 0.25 wt.% Ultrez 10 dispersion for 180 min (SD180) before proceeding to neutralization to form the microgel (SDM180). Figure 4 displays the steady-state flow curve of such dispersion before and after sonication. Both flow curves exhibit a slight non-Newtonian behavior characterized by power–law relationships ($\sigma =m{\dot{\gamma }}^{n}$ where *σ* is the shear stress, *m* is the consistency index, $\dot{\gamma }$ is the shear rate, and *n* is the shear rate sensitivity index). It can be observed that SD180 is a little less viscous than the non-sonicated one (SD0), as expected, because of possible ultrasound damage on the Ultrez 10 macromolecules, which would result in a decrease in its *M_w_* (see discussion below). Concomitantly, a softening effect of the microgel microstructure is expected. Measurements of the pH at the same temperature in the dispersion before and after sonication were 2.40 ± 0.03, which indicates that ultrasound treatment does not influence the amount of ionized carboxyl groups in the dispersion. 

**Figure 5:**
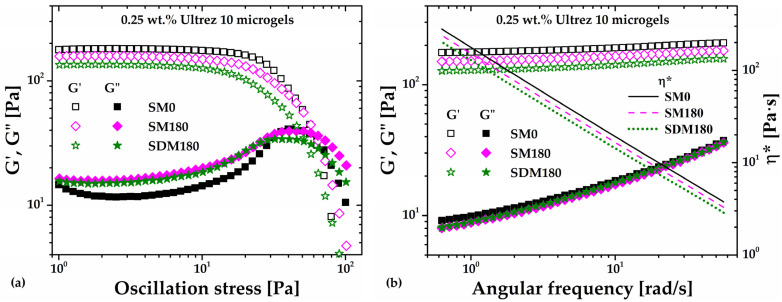
(**a**) Stress amplitude sweep at 6.28 rad/s and (**b**) frequency sweep at 3 Pa for the Ultrez 10 microgel obtained from the sonicated precursor dispersion (SDM180). The sweeps corresponding to the SM0 and SM180 microgels are included for comparison.

Figure 5a,b show the stress amplitude sweep at an angular frequency (*ω*) of 6.28 rad/s and the frequency sweep at 3 Pa, respectively, for the microgel (SDM180) obtained from the SD180 dispersion (the sweeps corresponding to the SM0 and SM180 microgels are included for comparison and η* is included in Figure 5b for completeness). The slight decrease in the *G′* value is apparent for SDM180 in comparison with the SM180 sample, while the *G″* value does not change. If ultrasound irradiation breaks polyacrylic acid macromolecules, the small decrease in *G’* could be attributed to the formation of the SDM180 microgel from shorter molecules, i.e., with reduced molecular weight, leading to a slightly softer microgel structure than the SM180 microgel, which was composed of raw Ultrez 10. This result would agree with reports suggesting that stronger hydrogels result from increasing the degree of cross-linking (see Figure 6 in [21]) and/or the molecular weight of the polymer [22].

### 2.4. Molecular Weight Measurements and FTIR of Ultrez 10 before and after Sonication

Molecules dissolved in water can undergo pyrolysis and free radical attack when submitted to sonication [4,23]. Also, macromolecules may be broken in their main chain due to sonication [23]. The exact mechanism leading to chain scission is still not fully clear. One of the most accepted theories is that the shear forces generated by the rapid motion of the solvent on cavitation collapse are responsible for the breakage of the chemical bonds within the polymer [24]. Another mechanism suggests, however, that there is an elongational field, rather than a shear field, during bubble collapse [25]. In any event, bond scission occurs near the center of gravity of the macromolecules [25,26] due to the high shear/elongation rates generated in the process. 

Different authors have reported the degradation of polymer molecules when submitted to sonication. For example, Schittenhelm and Kulicke [25] used ultrasonic degradation to create a homologous series of molar masses for establishing structure–property relationships in cellulose derivatives. These authors reported a diminution of molecular mass and polydispersity for hydroxyethylsulfoethyl cellulose with increasing the sonication time. More recently, Zhong et al. [27] reported a decrease in the average molecular weight of schizophyllan and degradation products with a narrower molecular weight distribution after ultrasonic treatment. Also, the original non-Newtoninan shear-thinning behavior of untreated schizophyllan changed to a Newtonian one for the resulting fractions. To investigate the effect of ultrasound on the molecular structure of Ultrez 10 and its effect on microgel formation in this work, weight-average molecular weight (*M_w_*) measurements using static light scattering (SLS) and Fourier transform infrared spectroscopy (FTIR) were performed on the raw and sonicated polymer as shown below. 

**Figure 6:**
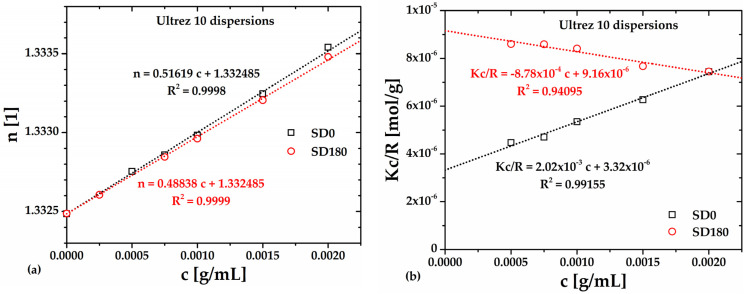
(**a**) *n* and (**b**) *Kc*/*R_θ_* as functions of Ultrez 10 concentration.

To determine the *M_w_* by SLS, the refractive index, *n*, of Ultrez 10 dispersions is measured as a function of its concentration, *c*; the results are presented in Table 2 and plotted in Figure 6a, respectively, for the SD0 and SD180 dispersions. The d*n*/d*c* values for each sample are obtained by linear fitting of the data, and the resulting equations are embedded in the Figure 6a. These values are used to calculate the optical constant, *K*, which is necessary to obtain the *Kc*/*R_θ_* values, also presented in Table 2 and plotted in Figure 6b versus *c*. The Rayleigh’s ratios, *R_θ_*, are calculated using the refractive indexes of water and toluene as well as the Rayleigh’s ratio of toluene.

In Figure 6b, the dotted lines indicate the fittings to the Debye equation for SD0 and SD180. *M_w_* and *A*_2_ are calculated from the ordinate to the origin and the slope of each linear relationship for SD0 and SD180 samples, respectively; the resulting values are reported in Table 3. Clearly, the lowest *M_w_* corresponds to the SD180 sample, which is less than half the value corresponding to SD0; this reflects the effect of ultrasound treatment on the molecular structure of Ultrez 10. Interestingly, *A*_2_ changes from positive for SD0 to negative for SD180, indicating that polymer-solvent interactions are favored before sonication; meanwhile, polymer–polymer interactions are promoted after sonication [28].

**Figure 7:**
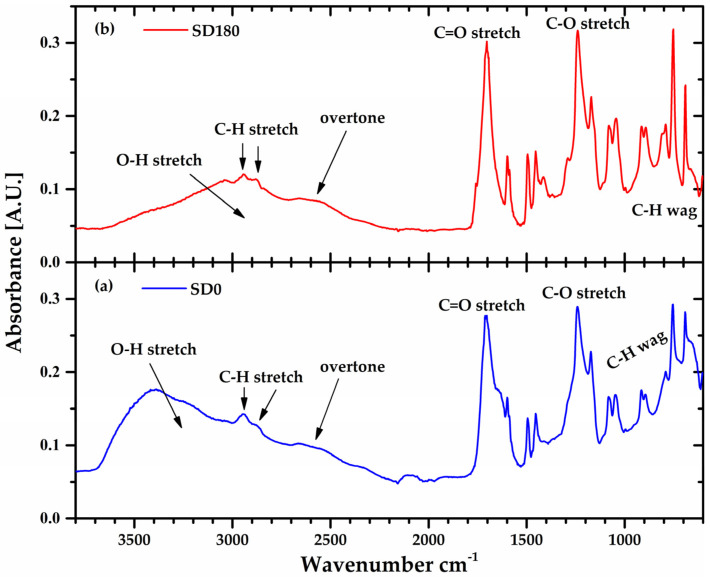
FTIR spectra of the lyophilized Ultrez 10 from the (**a**) non-sonicated (SD0) and (**b**) sonicated (SD180) precursor dispersions.

The change in sign in *A*_2_ for the sonicated polymer (Figure 6b) has also been related to a change in the chemical structure of the macromolecule due to depolymerization induced by prolonged periods of sonication [11,29,30,31,32,33,34]. Figure 7 shows the Fourier transform infrared (FTIR) spectra of the lyophilized Ultrez 10 obtained from the SD0 and SD180 dispersions, respectively. For both samples, the peaks centered around 1705 and 1240 cm^−1^, corresponding to the stretching of C=O and C-O bonds, respectively, along with the broad band from 3700 to 2400 cm^−1^, indicating the presence of carboxylic groups. In particular, the small band between 2700 and 2400 cm^−1^ indicates dimer formation (overtone) or hydrogen bonding, which is characteristic of the polyacrylic acid in its solid (powder) state. In addition, the peaks located at low wavenumbers (<1000 cm^−1^) represent the wagging of C-H, which indicates the presence of C=C. Thus, the increase in the height of these peaks for SD180 suggests the scission of the C-C links in the backbone and the concomitant formation of C=C bonds at the ends of the new shorter and less polar polymer chains, which is consistent with the reduction in the molecular weight of Ultrez 10 and the change in sign of *A*_2_. These results are consistent with previous reports on the effect of sonication on the molecular structure of other water-soluble polymers [27,32,34]. In addition, the reduction in the *M_w_* of the Ultrez 10 in the aqueous solutions and microgels, as well as their change in the rheological properties, agree well with that induced by ultrasound treatment for schizophyllan [27], water-soluble polymers [32] and sodium alginate [34].

**Figure 8:**
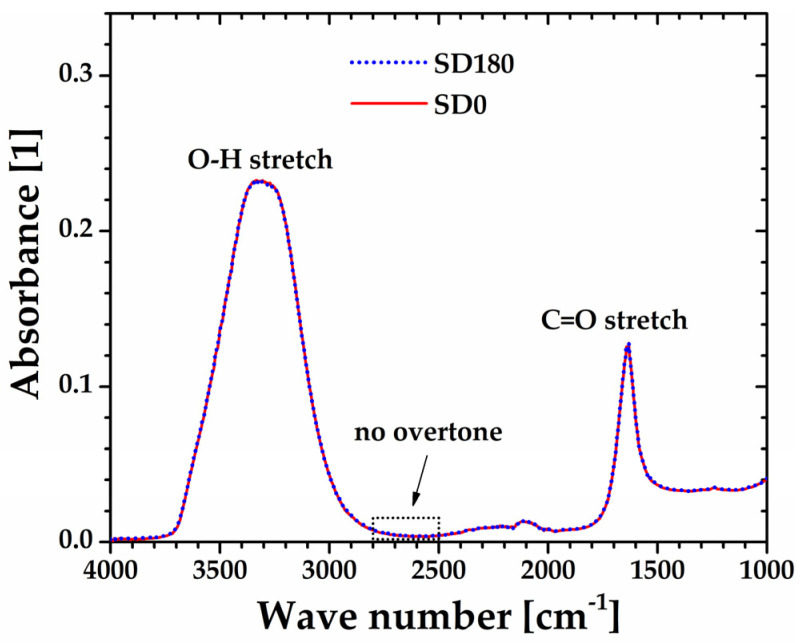
FTIR spectra of the non-sonicated (SD0) and sonicated (SD180) precursor dispersions.

On the other hand, Figure 8 shows the FTIR spectra of the sonicated (SD180) and non-sonicated (SD0) aqueous dispersions, which appear remarkably well superimposed. The broad band from 3700 to 2900 cm^−1^, along with the peak centered at 1700 cm^−1^ attributed to O-H and C=O stretchings, respectively, are characteristic of carboxylic acid groups present along the backbone of polyacrylic acid. More importantly, the lack of a band for both SD0 and SD180 samples from 2800 to 2500 cm^−1^, typical of carboxylic acids in their solid and liquid state, is indicative of complete dissociation of the carboxylic acid groups [32], which is consistent with the pH = 2.40 ± 0.03 measured for both the SD0 and SD180 samples. Thus, this result suggests that the softening of the microgel may be attributed only to a decrease in the molecular weight of Ultrez 10 macromolecules due to depolymerization occurring in their main backbone.

**Figure 9:**
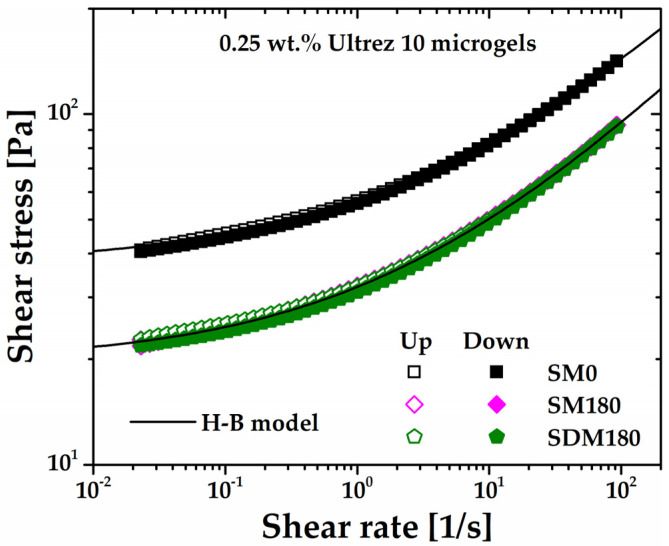
Up and down flow curves of the microgel SDM180. The flow curves corresponding to the SM0 and SM180 microgels are included for comparison.

A separate test of similarity between SM180 and SDM180 microgels is obtained from steady-state flow measurements. Figure 9 shows the up and down flow curves of the SDM180 microgel as compared to SM0 and SM180 ones. First, note that the SDM180 and SM180 exhibit almost the same flow behavior, i.e., they are very well described by the same H-B model. Then, the up and down SDM180 flow curves superimpose with the corresponding SM180, indicating that ultrasound treatment before gel formation does not induce thixotropy either. Interestingly, this is consistent with the viscous response obtained from the oscillatory measurements in Figure 5, where the *G*″ values are similar for the SM180 and SDM180 microgels. Thus, these results further demonstrate that sonication of the precursor dispersion (that results in the SDM180 microgel) or the already formed gel (SM180) produces softer microgels as compared to the non-sonicated one, with SM180 and SDM180 showing similar shear rheological properties (see Figure 9).

Although our study was carried out at constant power, it is expected that increasing the sonication power and time (this last implicitly introduces more energy) results in a decrease in the rheological properties. There may be a limit to this decrease, provided there is a limiting degree of depolymerization [24]. Also, further input of energy by increasing power and time of sonication may result in the collapse of the 3D network comprising the gel microstructure.

### 2.5. Confocal Microscopy of Non-Sonicated and Sonicated Ultrez 10 Microgels

**Figure 10:**
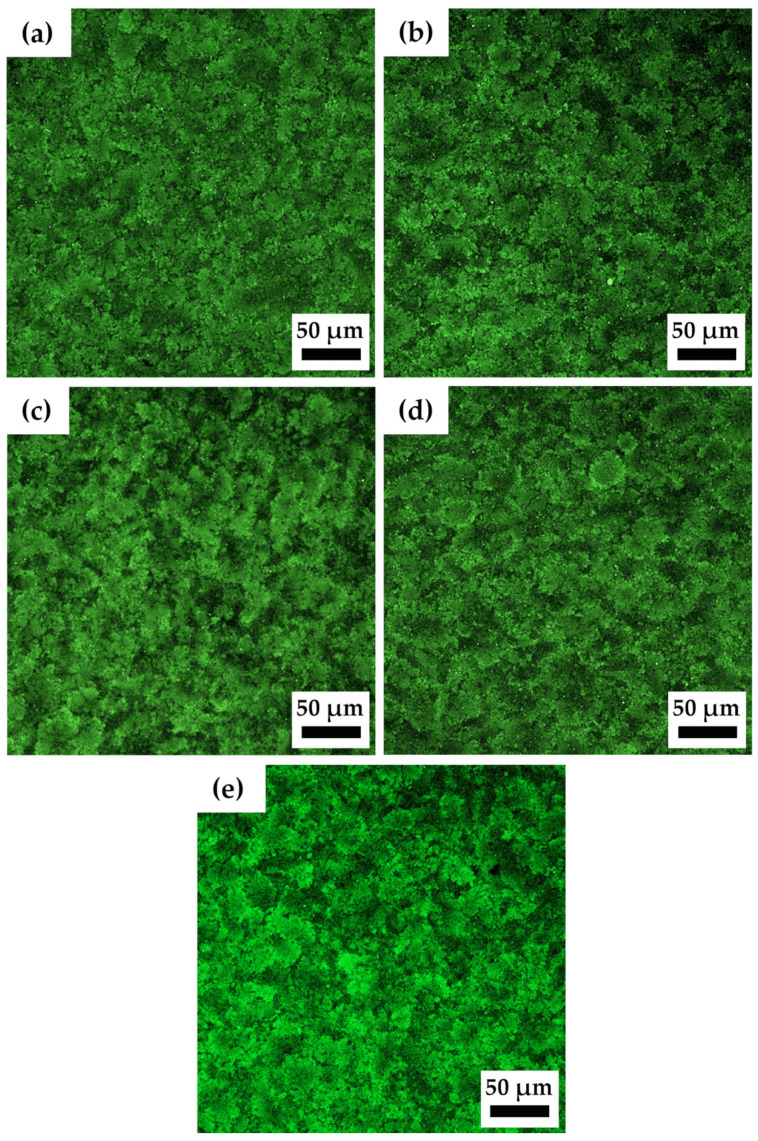
Confocal images of Ultrez 10 microgels with different sonication times: (**a**) SM0, (**b**) SM60, (**c**) SM120, (**d**) SM180 and (**e**) SDM180.

Confocal microscopy observations of the non-sonicated and sonicated Ultrez 10 microgels were performed to evidence changes in their microstructure due to sonication. Figure 10a–e show the resulting characteristic microstructure of SM0, SM60, SM120, SM180 and SDM180 microgels, respectively. It can be observed that microgel microstructure becomes more open or less compact with increasing the sonication time, as well as for the SDM180 sample. The domains [35] appear to increase in size with sonication time, leaving a less compact or more porous structure with more free solvent. Interestingly, the structure of the microgels with longer sonication time resembles that of less concentrated microgels (see, for example, [18,36]). Oelschlaeger et al. [36] analyzed the microstructure of Ultrez 10 microgels and its relationship with macroelasticity. These authors found that the bulk shear modulus, |G*|, strongly depends on the fraction of compact regions formed by aggregated particles in which less free solvent is available (see Figure 3 in [36]). It is expected that decreasing such compact regions and more free solvent with increasing the sonication time results in enhanced lubrication among sponges and the concomitant decrease in viscoelastic characteristics of the microgel, these represented by the shear modulus, viscosity and yield stress, in agreement with results in Figure 1, Figure 2 and Figure 3. Also, the SLS measurements presented above corroborate the change in molecular weight of Ultrez 10 macromolecules due to sonication, which results in softer microgels.

## 3. Conclusions

The effect of sonication on the molecular structure of polyacrylic acid (Carbopol^®^ Ultrez 10) and its rheological behavior in aqueous dispersions and microgels containing 0.25 wt.% of the polymer was analyzed in this work by rheometry, molecular weight measurements via static light scattering (SLS), Fourier transform infrared (FTIR) spectroscopy and confocal microscopy. For this, the precursor dispersion and the microgel were sonicated in a commercial ultrasound bath at a fixed power and at various times. The shear modulus, yield stress and viscosity of the microgel decreased systematically with an increase in the sonication time, reflecting a softening of the microgel microstructure. Meanwhile, the overall rheological behavior remained Herschel–Bulkley-like.

SLS measurements evidenced a reduction in the molecular weight of polyacrylic acid with sonication time; this reduced to almost one-third of its original value after 180 min. FTIR measurements show that sonication produces scission in the C-C links of the Carbopol^®^ backbone, which results in chains with the same chemistry but lower molecular weight. Confocal microscopy measurements revealed a diminution of the size of the microsponge domains with increasing sonication time, which is reflected in a softer microstructure resulting from the reduction in the molecular weight of polyacrylic acid. Finally, the results in this work indicate that both the microstructure and rheological behavior of microgels, in particular, and complex fluids, in general, may be manipulated or tailored by high-power ultrasonication. Special interest may be paid to naturally occurring or synthetic polymers whose high molecular weight results in high-viscosity solutions and gels that limit their applications. Future work considers additional characterization of the microgels after being submitted to variable ultrasound power, as well as its application to other gel-forming polymers of practical interest.

## 4. Materials and Methods

The polymer utilized in this work was a polyacrylic acid, Carbopol^®^ Ultrez 10 (Lubrizol Corporation, Wickliffe, OH, USA). Carbopol^®^ resins are hydrophilic cross-linked acrylic acid polymers differing in cross-link density. The more highly cross-linked members of the Carbopol^®^ family are rigid particles, while the more lightly cross-linked members are delivered as micron-sized powder particles, which can largely swell, being these last best representatives of microgels [37]. When the resin is mixed with water, an acid dispersion is obtained. Upon neutralization with a suitable base, the protons in the carboxylate groups are substituted by the cation of the base, and the molecules adopt a highly expanded configuration. The as-formed, highly swollen and deformable particles resemble individual sponges that give rise to elastoviscoplastic microgels [35].

The structure and rheological behavior of Carbopol^®^ dispersions and microgels are dependent on their preparation conditions, including the type of mixing, mixing procedure and pH [20,38]. Excessive shear during mixing affects the microstructure and rheological behavior of the as-formed microgels. Particularly, excessive shear of Carbopol^®^ Ultrez 10 microgels has been reported to produce thixotropy [15]. Therefore, dispersions and microgels in this work were prepared, respectively, following a standard procedure to obtain non-thixotropic or simple yield-stress microgels [14,17,39]. For this, Carbopol^®^ Ultrez 10 was dissolved at 0.25 wt.% in bi-distilled water under continuous stirring at 500 rpm for 1 h with a twisted three-blade turbine impeller. Since Carbopol^®^ microgels are sensitive to fungus and bacteria, we added 0.5 wt.% of phenoxyethanol as a preservative to the dispersion to ample the microgel’s useful lifetime from a few days up to two weeks. (It is noteworthy here that after this lapse, the microgels start to age even though they do not show evidence of fungus and/or bacteria for several months.) Then, the dispersion containing preservative was neutralized with a 5 mol/L NaOH aqueous solution to obtain a pH = 7.02 ± 0.02 while keeping the same stirring conditions until the gel was well formed and free of air bubbles (0.5 h). It is known that the rheological behavior of Carbopol microgels is very sensitive to pH, with a broad maximum or plateau at around pH 5–10, and there is a considerable decrease in the yield stress and viscosity out of this range [18,40]. In this work, we used the neutral pH since it produces a jammed structure of swelled sponges that raises the characteristic gel behavior [35]. Once prepared, the microgel sample was maintained at rest in a dark place at ambient temperature for one day. Afterward, beakers containing 70 mL of microgel were subjected to sonication for 0, 60, 120 and 180 min, respectively, in a water-containing ultrasonic cleaner (Cole-Parmer^®^, Vernon Hills, IL, USA) of 1.5 L of capacity and 150 Watts of sonic power. Sonication started at room temperature (~26 °C), which increased up to 63 °C after 180 min due to the energy dissipated by the ultrasonic treatment. This temperature rise does not affect the molecular characteristics of Ultrez 10 aqueous dispersions and microgels since thermal depolymerization of polyacrylic acid aqueous solutions occurs at above 180 °C and high pH conditions (~10) [41]. The samples with different sonication times are labeled as sonicated microgels: SM0, SM60, SM120 and SM180, respectively.

For comparison, another microgel was prepared from a previously sonicated dispersion. In this case, a dispersion containing the same concentration of Ultrez 10 (0.25 wt.%) was prepared as stated above and left to rest for one day. Next, the dispersion was divided into two parts, and one of these submitted to 180 min of sonication. Then, the sonicated dispersion was neutralized to obtain another microgel sample labeled as sonicated dispersion microgel: SDM180.

### 4.1. Rheological Characterization

The rheological behavior of the microgels was assessed via small-amplitude oscillatory shear (SAOS) and steady shear measurements using an AR G2 stress-controlled rheometer (TA Instruments, New Castle, DE, USA) and the parallel-plate geometry. Since this type of microgel is very prone to slip at the shearing surfaces, the plates were covered with sandpaper # 150 to suppress slip [14]. As for the neat dispersions (with and without sonication), their flow behavior was analyzed by using a double-gap Couette cell attached to the AR G2 rheometer. All the flow experiments were performed at 25 °C; for this, the rheometer was equipped with Peltier systems for temperature control of both the parallel-plate geometry and double-gap cell. Finally, despite the fact that this microgel preparation procedure resulted in reproducible rheological behavior for up to two weeks [39], all steady and dynamic flow experiments were carried out using fresh samples and preceded by a conditioning pre-shear of the microgel at 100 s^−1^ for 60 s, followed by one minute at rest to eliminate any possible aging affect [42] and to start the rheological experiments with similar gel microstructure [15]. 

### 4.2. Determination of Weight-Average Molecular Weight (M_w_) of Ultrez 10 

To assess possible changes in the molecular characteristics of Carbopol^®^ Ultrez 10 due to sonication, its *M_w_* and the second virial coefficient (*A*_2_) before and after sonication were determined at a temperature of 25 °C by static light scattering (SLS, Litesizer™ 500, Anton Paar GmBH, Graz, Austria). The SLS technique was chosen as it allows the measurement of absolute *M_w_* [43] and has been successfully used to measure the *M_w_* of other polyelectrolyte molecules [44]. *M_w_* and *A*_2_ are obtained from the following:

(1) $$\frac{Kc}{R_{\theta }}=\frac{1}{M_{w}}+2A_{2}c$$

where *K* is the optical constant, *c* is the concentration of Carbopol^®^ Ultrez 10 in the solution, and *R_θ_* is the Rayleigh ratio. The *K* and *R_θ_* were calculated as follows [38]:

(2) $$K=\frac{2\pi^{2}}{\lambda^{4}N_{A}}{\left(n_{0}\frac{dn}{dc}\right)}^{2}$$

(3) $$R_{\theta }=\frac{(I_{s}-I_{0})n_{0}^{2}}{I_{T}n_{T}^{2}}R_{T}$$

In Equation (2), *λ* = 658 nm is the wavelength of the incident light, *N_A_* is Avogadro’s number, *n*_0_ = 1.332485 is the refractive index of solvent (bidistilled water), and d*n*/d*c* is the rate of change in the refractive index as a function of concentration. In Equation (3), *I*_S_ and *I*_0_ are the scattered light intensities of solutions and solvent, respectively; *I_T_* is the dispersed light intensity of a standard (toluene in this case); *n_T_* = 1.48983 is the refractive index of toluene; and *R_T_* = 1.14574 × 10^−5^ cm^−1^ is the Rayleigh’s ratio of toluene.

To measure the molecular weight of the Ultrez 10 before and after sonication, six aqueous dissolutions were prepared from the sonicated (SD180) and non-sonicated (SD180) dispersions, both with an initial concentration of 2.5 mg/mL. The dissolutions were prepared at 10, 20, 30, 40, 60 and 80%, for which the refractive index as a function of Ultrez 10 concentration was first determined using a refractometer (Abbemat 550, Anton Paar, Graz, Austria). For the refractive index determination, 0.3 mL of each sample measured with a micropipette was placed in the refractometer. The refractive index of the bidistilled water used to prepare the microgels and dissolution was then found to be *n =* 1.332485 (see Table 2). These data were used to compute the *Kc*/*R_θ_* values and afterward to determine the molecular weight using the SLS technique. For this, 1.2 mL of each dissolution was added into a clean cuvette of quartz, which was, in turn, inserted in the Litesizer to start measurements. All experiments were carried out at 25 °C.

### 4.3. Confocal Microscopy

The changes in microgel microstructure, along with sonication time, were assessed by confocal microscopy using an LSM 800 (Zeiss Group, Oberkochen, Germany) microscope with a green light excitation λ = 532 nm and the Efficient Navigation Software (version 2.6 Blue edition, Zeiss group, Oberkochen, Germany). For this, a few drops of a 1.1 × 10^−4^ M Rhodamine 6G (Sigma-Aldrich, St. Louis, MO, USA) aqueous solution [15] were added to the samples and gently mixed to obtain uniform red-colored microgels. Then, the microgel samples were placed on glass slides for observation. The micrographs were acquired with apochromatic objectives of 20× and 40× with numerical apertures of 0.8 and 1.3, respectively.

### 4.4. Fourier Transform Infrared Spectroscopy

The identification of the functional groups of the non-sonicated and sonicated lyophilized Ultrez 10 samples was carried out by Fourier transform infrared spectroscopy (FTIR) using a IRAffinity spectrometer (Shimadzu, Kyoto, Japan) with an Attenuated Total Reflection (ATR) accessory in the absorbance mode, and in the wavenumber range from 600 to 3800 cm^−1^, with 4 cm^−1^ resolution, 16 scans. To obtain the FTIR spectrum of the Carbopol^®^ Ultrez 10 before and after sonication, the dispersions SD0 and SD180 were dried by lyophilization. For this, 100 mL of the dispersion was placed in a lyophilization vessel, frozen at −10 °C, and then introduced into a lyophilizer (FreeZone 6, LABCONCO, Kansas City, MO, USA). Freeze drying was carried out for eight hours/day for three days at a vacuum pressure of 7 × 10^−3^ mBar and a temperature of −25 °C. At the end of each day, the vessel was placed in a freezer to continue the process the next day. Then, 1 mg of the lyophilized Ultrez 10 powders was placed and gently pressed against a high-refractive-index diamond prism located in the ATR accessory to measure the infrared spectrum.

## Figures and Tables

**Table 1:** Herschel–Bulkley parameters of 0.25 wt.% Ultrez 10 microgels sonicated at various times.

Microgels	*σ_y_* [Pa]	*m* [Pa·s^n^]	*n* [1]
SM0	37.2	19.2	0.37
SM60	31.6	16.0	0.38
SM120	26.4	14.0	0.37
SM180	19.6	12.6	0.39
SMD180 *	20.5	11.7	0.40

* The values for SMD180 correspond to a microgel that is prepared using the precursor dispersion sonicated for 180 min; its rheological behavior is discussed in Section 2.4.

**Table 2:** Concentration of Ultrez 10 dispersions (*c*), *n*, and *Kc*/*R_θ_* for *M_w_* determination.

Sample	*c* [mg/mL]	0.00	0.25	0.50	0.75	1.00	1.50	2.00
SD0	*n* [1]	1.332485	1.332651	1.332753	1.332858	1.332983	1.333245	1.333540
	*Kc*/*R_θ_* [mol/g]	--	--	4.4712 × 10^−6^	4.703 × 10^−6^	5.35064 × 10^−6^	6.26993 × 10^−6^	7.44143 × 10^−6^
SD180	*n* [1]	1.332485	1.332605	1.332699	1.332845	1.332961	1.333205	1.333480
	*Kc*/*R_θ_* [mol/g]	--	--	8.6022 × 10^−6^	8.5915 × 10^−6^	8.41236 × 10^−6^	7.67077 × 10^−6^	7.44605 × 10^−6^

Measurements of *n* and *Kc*/*R_θ_* were obtained at 25 °C by using a refractometer and SLS technique (Litesizer™ 500).

**Table 3:** *M_w_* and *A*_2_ of the Ultrez 10 before and after 180 min of sonication.

SD0		SD180	
*M_w_* [g/mol]	*A*_2_ [mol·cm^3^/g^2^]	*M_w_* [g/mol]	*A*_2_ [mol·cm^3^/g^2^]
300,860	1.0102 × 10^−3^	109,212	−4.3999 × 10^−4^

The data in this table were obtained at 25 °C by using a refractometer and SLS technique (Litesizer™ 500).

## Data Availability

The raw/processed data required to reproduce these findings are available upon request.
